# Modulation of NCC activity by low and high K^+^ intake: insights into the signaling pathways involved

**DOI:** 10.1152/ajprenal.00255.2013

**Published:** 2014-04-23

**Authors:** María Castañeda-Bueno, Luz Graciela Cervantes-Perez, Lorena Rojas-Vega, Isidora Arroyo-Garza, Norma Vázquez, Erika Moreno, Gerardo Gamba

**Affiliations:** ^1^Molecular Physiology Unit, Instituto Nacional de Ciencias Médicas y Nutrición Salvador Zubirán and Instituto de Investigaciones Biomédicas, Universidad Nacional Autónoma de México, Tlalpan, Mexico City, Mexico; and; ^2^Departamento de Farmacología, Instituto Nacional de Cardiología Ignacio Chávez, Tlalpan, Mexico City, Mexico

**Keywords:** with-no-lysine kinase 4, Ste20-related proline-alanine-rich kinase, aldosterone, distal convoluted tubule

## Abstract

Modulation of Na^+^-Cl^−^ cotransporter (NCC) activity is essential to adjust K^+^ excretion in the face of changes in dietary K^+^ intake. We used previously characterized genetic mouse models to assess the role of Ste20-related proline-alanine-rich kinase (SPAK) and with-no-lysine kinase (WNK)4 in the modulation of NCC by K^+^ diets. SPAK knockin and WNK4 knockout mice were placed on normal-, low-, or high-K^+^-citrate diets for 4 days. The low-K^+^ diet decreased and high-K^+^ diet increased plasma aldosterone levels, but both diets were associated with increased phosphorylation of NCC (phospho-NCC, Thr^44^/Thr^48^/Thr^53^) and phosphorylation of SPAK/oxidative stress responsive kinase 1 (phospho-SPAK/OSR1, Ser^383^/Ser^325^). The effect of the low-K^+^ diet on SPAK phosphorylation persisted in WNK4 knockout and SPAK knockin mice, whereas the effects of ANG II on NCC and SPAK were lost in both mouse colonies. This suggests that for NCC activation by ANG II, integrity of the WNK4/SPAK pathway is required, whereas for the low-K^+^ diet, SPAK phosphorylation occurred despite the absence of WNK4, suggesting the involvement of another WNK (WNK1 or WNK3). Additionally, because NCC activation also occurred in SPAK knockin mice, it is possible that loss of SPAK was compensated by OSR1. The positive effect of the high-K^+^ diet was observed when the accompanying anion was citrate, whereas the high-KCl diet reduced NCC phosphorylation. However, the effect of the high-K^+^-citrate diet was aldosterone dependent, and neither metabolic alkalosis induced by bicarbonate, nor citrate administration in the absence of K^+^ increased NCC phosphorylation, suggesting that it was not due to citrate-induced metabolic alkalosis. Thus, the accompanying anion might modulate the NCC response to the high-K^+^ diet.

renal k^+^ excretion is dependent on K^+^ secretion in the distal nephron because the filtered K^+^ is almost completely reabsorbed in the proximal convoluted tubule. In the distal nephron, the Na^+^ reabsorption that occurs through the epithelial Na^+^ channel generates a lumen negative potential that serves as the driving force for K^+^ secretion through the renal outer medullary K^+^ (ROMK) channel. In addition, the rate of luminal flow is associated with the activation of large-conductance Ca^2+^-activated K^+^ channels (13, 15). Thus, K^+^ secretion in the distal nephron is a flow-dependent process that is coupled to Na^+^ reabsorption.

Salt reabsorption in the distal convoluted tubule (DCT) occurs through the thiazide-sensitive Na^+^-Cl^−^ cotransporter (NCC), which plays a key role in modulating salt and fluid delivery to downstream portions of the nephron and, thus, in K^+^ secretion. Two inherited diseases, which are the consequence of the elimination or activation of NCC, produce opposite effects on plasma K^+^ concentration: Gitelman's disease features hypokalemia, which is due to inactivating mutations in the gene encoding NCC (*Slc12a3*), and familial hyperkalemic hypertension or pseudohypoaldosteronism type II, a disease that features hyperkalemia, which may be the consequence of NCC activation by altered activity of mutant kinases or ubiquitin ligases, because it can be abrogated by either thiazide-type diuretics (19, 40) or the elimination of NCC in genetically engineered mice (17). Thus, decreased or increased NCC activity promotes K^+^ secretion or retention, respectively.

Therefore, it is not unexpected that the amount of ingested K^+^ modulates NCC expression and/or activity. Vallon et al. (34) observed that mice fed a low-K^+^ diet exhibited a significant increase in activating phosphorylation of NCC, an effect that would be expected to decrease Na^+^ and fluid delivery to the distal nephron, negatively affecting K^+^ secretion. In contrast, mice fed a high-K^+^ diet exhibited no to moderate decreases in NCC phosphorylation compared with the levels of phosphorylation observed in mice fed a normal diet. In another study, Frindt and Palmer (10) observed a moderate decrease in NCC apical expression in rats maintained on a high-K^+^ diet. In this regard, it is important to note that a high-K^+^ diet is a strong stimulus for increasing the synthesis and secretion of the mineralocorticoid hormone aldosterone, which is known to promote increased NCC expression (16). The increase in NCC expression is due, at least in part, to increased activity of serum/glucocorticoid regulated kinase 1 (SGK1), which, in turn, inhibits the ubiquitylation of NCC by the E3 ubiquitin ligase Nedd4-2 (1, 28). In addition, independent of its effect on NCC expression, aldosterone promotes NCC phosphorylation (35, 36). Thus, the explanation for the decreased phosphorylation and surface expression of NCC observed in mice fed a high-K^+^ diet in Vallon et al.'s and Frindt and Palmer's works remains elusive. However, Vallon et al. (34) observed that these effects were exacerbated in SGK1-deficient mice, possibly due to loss of the aldosterone-induced positive effect on NCC.

A low-salt diet increases and a high-salt diet decreases NCC activity (for reviews, see Refs. 5 and 11). We have shown that increased NCC and Ste20-related proline-alanine-rich kinase (SPAK) phosphorylation during maintenance on a low-salt diet or administration of ANG II does not occur in with-no-lysine kinase 4 (WNK4) knockout (WNK4^−/−^) mice, suggesting that the salt-modulated NCC regulatory pathway requires integrity of the WNK4-SPAK binomium (4, 29). Therefore, the present study was designed to analyze the roles of WNK4 and SPAK in K^+^-induced changes in NCC expression and phosphorylation, taking advantage of recently developed total WNK4^−/−^ (4) and SPAK knockin (SPAK^T243A/T243A^) (25) mouse models. In our study, both low- and high-K^+^ diets were associated with increased NCC phosphorylation. The observed effect of a high-K^+^ diet was aldosterone dependent. The increase in NCC phosphorylation in mice fed with both diets was correlated with an increase in SPAK phosphorylation and occurred in WNK4^−/−^ mice, suggesting that, in contrast to a low-salt diet, the response of NCC to changes in K^+^ intake is a WNK4-independent process.

## METHODS

### 

#### Animal experiments.

All experiments involving animals were approved by the Animal Care and Use Committee of our institution. For this work, we used two genetically altered mouse models that have been previously described: a WNK4^−/−^ mouse strain (4) and a SPAK knockin mouse strain (25). For experiments conducted with each of these strains, the wild-type controls were littermates of the homozygous mice. Briefly, in WNK4^−/−^ mice (4), WNK4 expression was knocked out by replacing exon 1 with a neomycin phosphotransferase cassette. In the SPAK knockin mouse strain (25), Thr^243^, which is required for WNK-induced activation of SPAK, was replaced with alanine (SPAK^T243A/T243A^). The genetic background of both strains was C57BL/6. Genotyping was performed as previously described (4, 25). Male mice between 12 and 16 wk old were used.

#### Low-K^+^, control, and high-K^+^ diets.

Control (1.2% K^+^), low-K^+^ (0% K^+^), and high-K^+^ (5% K^+^) diets were obtained from TestDiet (St. Louis, MO) and were prepared by modifying the AIN-93M semipurified diet, as previously described (4). The 0% K^+^ diet was used as the base, and tribasic K^+^-citrate was added to generate the 1.2% K^+^ and 5% K^+^ diets. After a 2-day period of adapting to the 1.2% K^+^ powder diet, during which time mice were also allowed to adapt to the metabolic cages, the diet was changed to 0% or 5% K^+^ for some animals, whereas the control group continued to receive the 1.2% diet. On *days 1* and *4* after switching to these diets, mice were placed in metabolic cages for urine collection. At the end of *day 4*, mice were euthanized for urine and blood collection. The concentrations of urinary and plasma electrolytes were determined using a Synchron CX5 (Beckman Coulter, Miami, FL). The plasma aldosterone concentration was measured by ELISA (DRG), and plasma renin activity was measured by RIA (REN-CT2, RADIM). For only a particular experiment, K^+^ was added to the diet as KCl, to obtain a high-K^+^ diet (5%) with high Cl^−^ content instead of high citrate content.

#### Spironolactone treatment.

To determine whether the findings for mice fed high-K^+^ diets were due to a concomitant aldosterone increase, the mineralocorticoid receptor was blocked with spironolactone in mice placed in individual cages. Mice were given normal- or high-K^+^ diets and simultaneously treated with spironolactone (Sigma) that had been dissolved in ethanol at 25 mg/ml and then added to the drinking water, as previously described (21). The calculated dose was 40 mg·kg^−1^·day^−1^. Water intake was monitored daily.

#### HCO_3_^−^ loading.

Protocol and doses were adapted from previous reports (22, 38). After mice underwent a period of adaptation to metabolic cages, 0.28 M NaHCO_3_ + 1% sucrose was added to the drinking water of the alkalosis group, whereas 0.28 M NaCl + 1% sucrose was added to the drinking water of the control group to maintain Na^+^ intake at similar levels. Mice were fed ad libitum with standard pelleted chow. Twenty-four-hour urine collections were done on the days before the start of treatment and on *day 7*. At the end of the second collection, mice were euthanized and kidneys were collected.

#### Citrate diet.

Mice were maintained on normal chow and were given a drinking solution containing 100 mM citric acid and 0.5% sucrose for 4 days. NaOH was used to adjust the pH to 4. The amount of NaOH added was measured, and an equivalent amount of Na^+^ was added as NaCl to the drinking water of the control group (also containing 0.5% sucrose). Mice were kept in metabolic cages to monitor water intake, which was similar between groups. According to the observed water intake, the citrate intake in this experimental group was ∼70% of that observed in the high-K^+^-citrate group. This corresponds to seven times the amount of citrate contained in the normal diet (in the high-K^+^-citrate diet, the amount citrate content was 10 times higher).

#### ANG II infusion.

The effect of ANG II infusion on NCC phosphorylation in WNK4^−/−^ mice has been previously reported (4). In this study, we assessed the effect of ANG II in the SPAK knockin mouse strain using microosmotic pumps (model 1007, Alzet, DURECT) implanted subcutaneously to infuse ANG II at a rate of 280 ng·kg^−1^·min^−1^ (400 μg·kg^−1^·day^−1^) (4). It has been previously reported that this dose does not cause pressor effects (23). Two 24-h urine collections were performed on the first and last days of the 4-day infusion. After the last day of infusion, animals were euthanized and kidneys were collected.

#### Immunoblot assays.

Kidney proteins and testes proteins were extracted with a lysis buffer containing 50 mM Tris·HCl (pH 7.5), 1 mM EGTA, 1 mM EDTA, 50 mM sodium fluoride, 5 mM sodium pyrophosphate, 1 mM sodium orthovanadate, 1% (wt/vol) Nonidet P-40, 0.27 M sucrose, 0.1% (vol/vol) 2-mercaptoethanol, and protease inhibitors (Complete tablets, Roche). For the SDS-PAGE analysis, 50–80 μg of protein were loaded per lane. Proteins were transferred to polyvinylidene difluoride membranes, which were then blocked for 1.5 h in 10% (wt/vol) nonfat milk dissolved in Tris-buffered saline-Tween. Antibodies were diluted in Tris-buffered saline-Tween containing 5% (wt/vol) nonfat milk. Membranes were incubated with primary antibodies overnight at 4°C and with secondary antibodies at ambient temperature for 1.5 h. The immobilized antigens were detected by chemiluminescence using the Luminata Crescendo detection system from Millipore.

#### Phosphatase treatment of kidney protein samples.

One-half of a mouse kidney was lysed following the usual protocol, whereas the second half was prepared with lysis buffer lacking phosphatase inhibitors. Protein was quantified, 40 μg of protein of each sample were transferred to new tubes (∼2 μl of lysate), and samples were diluted ∼10 times with 1× PMP NEB buffer and 1 mM MnCl_2_. Four hundred units (1 μl) of lambda protein phosphatase (P0753, New England Biolabs) were added to the sample that was prepared without phosphatase inhibitors. Samples were incubated at 30°C for 30 min, and Western blot analysis was performed following the usual protocol.

#### Antibodies.

Polyclonal antibodies against NCC, SPAK, oxidative stress response kinase 1 (OSR1), and phosphorylated (p)SPAK at Ser^383^ (S-motif) were raised in sheep. The concentrations used were 1–3 μg/ml. These antibodies were produced at the MRC phosphorylation unit in Dundee University, and their specificity has been previously demonstrated (25, 26, 30). A sheep antibody against pNCC at Thr^44^, Thr^48^, and Thr^53^ (in the mouse) was used. This antibody was also produced in the MRC phosphorylation unit of Dundee University and has been previously used by others (14). However, since the characterization of this antibody has not been published, we confirmed the specificity by performing Western blots with samples of NCC^+/+^ and NCC^−/−^ mice (31) (see results). In addition, for all phospho-blots, antibody incubation was done in the presence of nonphosphopeptide, following the recommendation of the manufacturer. This confirms that the antibody is specific for the phosphorylated epitope. The goat anti-actin polyclonal antibody conjugated to horseradish peroxidase and the donkey anti-sheep antibody were purchased from Santa Cruz Biotechnology.

#### Statistical analysis.

Bands from different blots were scanned for densitometry. For the NCC blots, the nonspecific band observed slightly above the 130-kDa marker band was not included in the densitometric analysis. Statistical significance was defined as two-tailed *P* < 0.05, and results are presented as means ± SE. Differences between two groups were tested for significance using Student's *t*-test. Differences between three or more groups were tested for significance using one-way ANOVA with multiple comparisons using the Bonferroni correction.

## RESULTS

### 

#### NCC is activated by both low- and high-K^+^ diets.

Wild-type mice were fed diets with high or low K^+^ content for a period of 4 days. To assess changes in the activity of NCC, K^+^ was added to the diet as K^+^-citrate instead of KCl to keep the Cl^−^ intake constant. The physiological parameters of wild-type mice fed low- or high-K^+^ diets were modified as expected (Table 1). Variations in dietary K^+^ content did not affect food intake, and, thus, the average weights of the low-, normal-, and high-K^+^ groups were similar. In agreement with this finding, urinary Na^+^ and Cl^−^ excretion was also similar. In mice fed a low-K^+^ diet, urinary K^+^ excretion was effectively reduced (0.02 ± 0.003 vs. 0.63 ± 0.09 mmol/24 h on the normal diet, *P* < 0.00005) and plasma aldosterone was decreased (90.5 ± 36.2 vs. 232.3 ± 88.4 pg/ml on the normal diet, *P* < 0.001). In contrast, urinary K^+^ excretion increased with the high K^+^ diet (2.57 ± 0.24 vs. 0.63 ± 0.09 mmol/24 h on the normal diet, *P* < 0.05) and the expected increase in plasma aldosterone was also observed (866.01 ± 383.49 vs. 232.3 ± 88.4 pg/ml on the normal diet, *P* < 0.05). However, plasma Na^+^ and K^+^ concentrations did not change significantly during the study. Notably, urinary volume was increased in the high-K^+^ group compared with the normal-K^+^ group (5.2 ± 1.2 vs. 2.1 ± 0.98 ml/24 h, *P* < 0.00005), but plasma renin activity was not affected. Finally, high-K^+^ group developed metabolic alkalosis due to the high citrate intake, as revealed by the higher urinary pH values (9.07 ± 0.08 vs. 7.67 ± 0.3 on the normal diet, *P* < 0.001) and the higher plasma CO_2_ concentration (22.15 ± 2.06 vs. 14.44 ± 2.48 on the normal diet, *P* < 0.05).

**Table 1.** Physiological parameters of WNK4^+/+^ and WNK4^−/−^ mice on NKD, LKD, or HKD

	WNK4^+/+^ Mice		WNK4^−/−^ Mice	
	Means ± SE	*n*	Means ± SE	*n*
Food intake, g				
NKD	3.2 ± 0.5	8	3.0 ± 0.4	9
LKD	2.8 ± 1.1	9	2.9 ± 0.3	10
HKD	3.3 ± 0.9	8	3.5 ± 0.2	9
Water intake, ml				
NKD	7.3 ± 1.6	8	8.1 ± 3.3	7
LKD	8.9 ± 2.9	9	7.5 ± 1.9	9
HKD	14.0 ± 3.0^†^	7	16.1 ± 2.5^†^	9
Urinary volume, μl				
NKD	2.1 ± 1.0	8	2.7 ± 1.0	9
LKD	2.4 ± 1.0	9	2.4 ± 1.6	10
HKD	5.2 ± 1.2^†^	8	5.3 ± 0.5^†^	9
Weight, g				
NKD	26.8 ± 3.1	8	25.7 ± 2.2	9
LKD	25.5 ± 2.3	9	23.4 ± 1.9	10
HKD	22.6 ± 2.7	8	22.2 ± 1.5	9
Plasma aldosterone concentration, pg/ml				
NKD	232.33 ± 88.43	7	234.27 ± 96.38	10
LKD	90.48 ± 36.23^†^	9	89.09 ± 56.36^†^	10
HKD	866.01 ± 383.49^†^	8	1,181.89 ± 617.79^†^	8
Plasma renin activity, ng ANG I·ml^−1^·h^−1^				
NKD	13.64 ± 7.03	7	N.D.	
LKD	N.D.		N.D.	
HKD	13.93 ± 3.12	6	N.D.	
*Urine data*				
Na^+^, mmol/24 h				
NKD	0.20 ± 0.04	6	0.24 ± 0.02	6
LKD	0.19 ± 0.02	8	0.16 ± 0.02	6
HKD	0.17 ± 0.01	8	0.15 ± 0.01	6
K^+^, mmol/24 h				
NKD	0.63 ± 0.09	6	0.70 ± 0.06	6
LKD	0.02 ± 0.003^†^	8	0.03 ± 0.004^†^	6
HKD	2.57 ± 0.24^†^	8	2.02 ± 0.08^†^	6
pH				
NKD	7.67 ± 0.3	8	N.D.	
LKD	N.D.		N.D.	
HKD	9.07 ± 0.08^†^	8	N.D.	
*Plasma data*				
Na^+^, mM				
NKD	152.85 ± 1.55	8	154.04 ± 6.12	9
LKD	152.28 ± 2.47	9	152.57 ± 2.85	10
HKD	155.14 ± 2.05	8	156.33 ± 2.45	9
K^+^, mM				
NKD	3.99 ± 0.34	8	3.26 ± 0.46*	7
LKD	3.44 ± 0.60	9	2.03 ± 0.33*^†^	9
HKD	4.45 ± 1.42	7	4.31 ± 1.00^†^	9
CO_2_, mM				
NKD	14.44 ± 2.48	8	16.18 ± 3.34*	9
LKD	13.48 ± 2.13	9	17.77 ± 2.67*	10
HKD	22.15 ± 2.06^†^	8	26.52 ± 4.41^†^	9

Values are presented as means ± SE; the number of animals per group (*n*) is also shown. WNK4, with-no-lysine kinase 4; NKD, normal K^+^ diet; LKD, low-K^+^ diet; HKD, high-K^+^ diet. Urine collected on *day 4* of the treatment period was analyzed.

* *P* < 0.05 vs. WNK4^+/+^ mice on the same diet;

† *P* < 0.05 vs. NKD (same genotype).

**Fig. 1.**
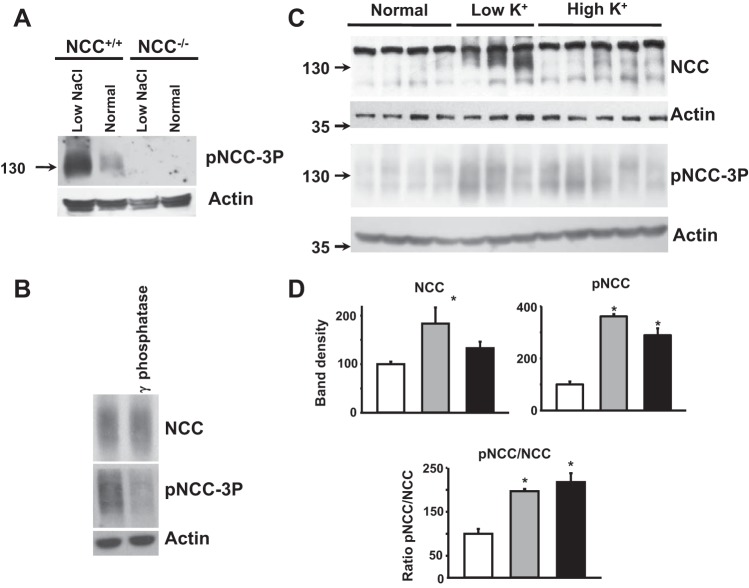
Effects of varying dietary K^+^ on Na^+^-Cl^−^ cotransporter (NCC) expression and phosphorylation. *A*: specificity of the NCC phospho-antibody used in this work. The antibody recognizes three phosphorylated residues in NCC (Thr^44^/Thr^48^/Thr^53^) [phosphorylated (p)NCC-3P]. Samples from wild-type (NCC^+/+^) and NCC-deficient (NCC^−/−^) mice kept on normal- or low-NaCl diets were analyzed by Western blot analysis. No signal was detected in lanes loaded with NCC^−/−^ samples, and signal intensity was greatly reduced when samples were treated with λ-phosphatase (*B*). The low signal observed in the phosphatase-treated sample was due to incomplete dephosphorylation. Nonphosphorylated peptide was included in the antibody solution for all blots against phosphorylated epitopes. *C*: representative Western blot analysis of total kidney protein samples of wild-type mice maintained on diets with normal, low, or high K^+^-citrate content. The solid band observed above the 130-kDa marker band in the NCC blots is a nonspecific band that was not included in the densitometric analyses. *D*: densitometric analyses were performed on at least two blots per assay, including samples of 6, 8, and 8 mice for the normal-K^+^ (open bars), low-K^+^ (shaded bars), and high-K^+^ (solid bars) diet groups, respectively. The average value in the control group was fixed as 100%, and the effect of the diet was normalized accordingly. Results are expressed as mean percentages ± SE of the normal diet (100%). *P < 0.001 vs. the normal diet.

To compare NCC expression and phosphorylation levels of the three groups, we performed Western blot analysis of total kidney protein extracts with a previously described NCC-specific antibody and a phospho-antibody recognizing NCC phosphorylated on Thr^44^, Thr^48^, and Thr^53^. The specificity of this antibody was confirmed by Western blot analysis of kidney protein samples from NCC^+/+^ and NCC^−/−^ mice. As shown in Fig. 1*A*, the signal was increased by the low-salt diet in wild-type mice and was not present in NCC^−/−^ mice fed either a regular or low-salt diet. In addition, protein treatment with alkaline phosphatase almost entirely eliminated the detected signal, indicating the requirement of phosphorylation for antibody recognition (Fig. 1*B*). These are sites phosphorylated by SPAK/OSR1, which are important for NCC activation under various conditions (23, 26). As previously reported by others (34), NCC phosphorylation increased with the low-K^+^ diet (Fig. 1, *C* and *D*). Despite the increase in total NCC, the pNCC-to-NCC ratio was higher in the low-K^+^ group than in the normal-K^+^ group, suggesting that the increase in phosphorylation levels was not only secondary to the increase in NCC expression.

**Fig. 2.**
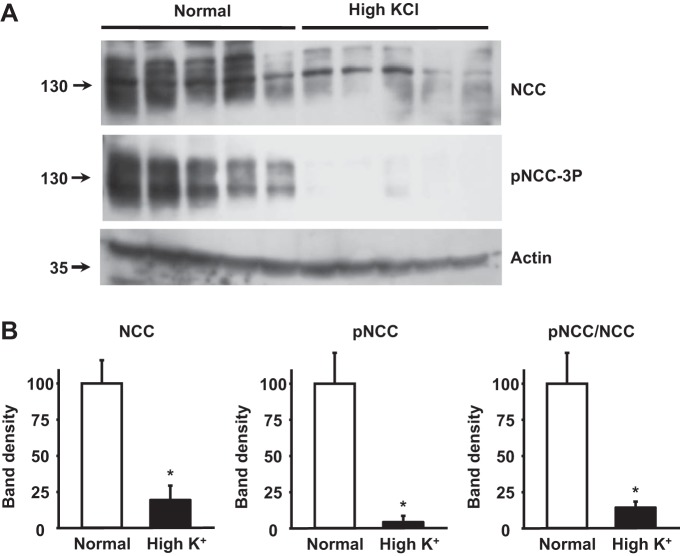
Effect of the high-KCl diet on the expression and phosphorylation of NCC. *A*: representative Western blot analysis of total kidney protein samples of wild-type mice maintained on diets with normal or high KCl content. *B*: densitometric analysis of blots shown in *A*. Results are expressed as mean percentages ± SE of the normal diet (100%). *P < 0.0001 vs. the normal diet.

**Fig. 3.**
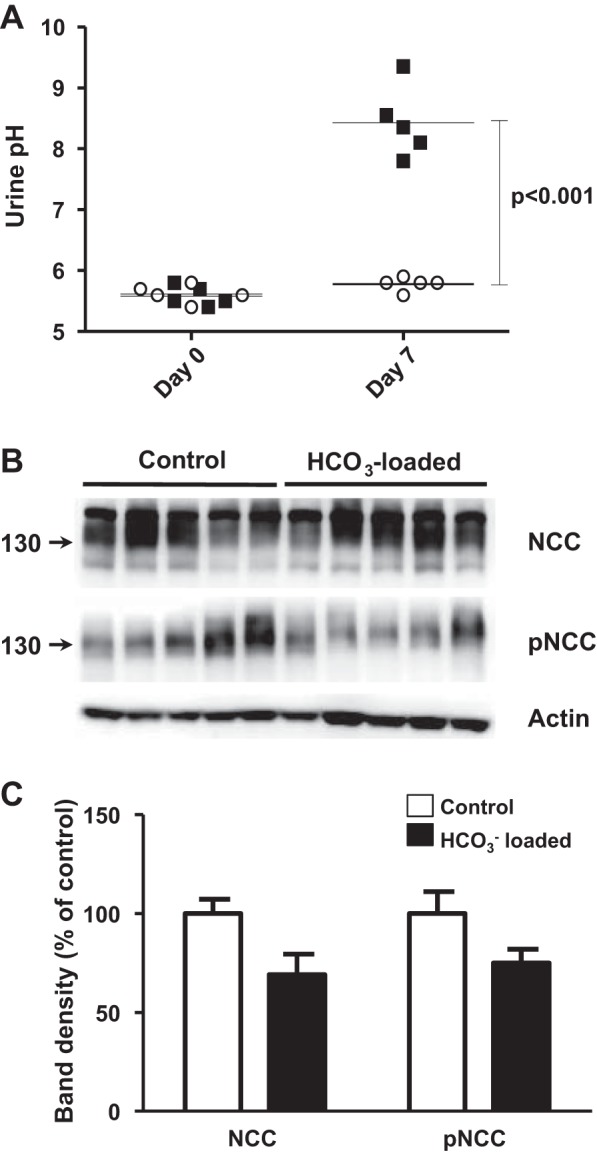
Effect of HCO_3_^−^ loading in expression and phosphorylation levels of NCC. *A*: urine pH of control mice (○) and HCO_3_^−^-loaded mice (☐) on the previous day to the beginning of treatment (*day 0*) and on *day 7* of high HCO_3_^−^ intake. The mean urine pH for each group is indicated by the horizontal line. *B*: Western blot analysis of kidney samples from control and HCO_3_^−^-loaded mice. *C*: densitometric analysis of the blot shown in *B*. No difference in NCC expression or phosphorylation was observed between groups.

**Fig. 4.**
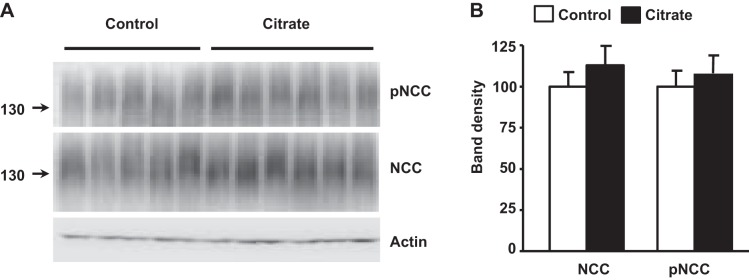
Effect of high citrate intake on NCC phosphorylation. *A*: Western blot analysis of renal proteins of wild-type mice fed with a high-citrate diet. Citrate was administered in the drinking water. Both groups consumed a similar amount of water (5.16 ± 1.6 ml for the control group vs. 5 ± 1.4 ml for the citrate group). *B*: results of the densitometric analysis expressed as mean percentages ± SE of control (100%). No significant difference was observed. Three blots per assay were included in the analysis.

Surprisingly, the high-K^+^ diet induced an increase in NCC phosphorylation (Fig. 1, *C* and *D*), in contrast to the mild decrease that has been previously reported in mice fed with a high-KCl diet (10, 34). In this study, we chose to administer K^+^ as K^+^-citrate to avoid possible confounding effects of high Cl^−^ intake. Indeed, when we fed mice with the high-KCl diet instead of high-K^+^-citrate diet, we were able to reproduce the decrease in NCC expression and phosphorylation previously observed (Fig. 2). As shown in Table 1, with the high-K^+^-citrate diet, mice developed a certain degree of metabolic alkalosis. It is unlikely, however, that the increased NCC phosphorylation was due to diet-induced metabolic alkalosis, because such an effect was not observed in wild-type mice in which metabolic alkalosis was induced through HCO_3_^−^ loading (Fig. 3). In addition, it is unlikely that citrate by itself was responsible since high citrate intake, without K^+^, did not stimulate an increase in NCC phosphorylation (Fig. 4). Thus, our data show that high K^+^-citrate intake increased, whereas high KCl intake decreased, NCC phosphorylation, suggesting that the coadministered anions may also play a role in NCC regulation.

#### Low-K^+^ diet promotes SPAK phosphorylation independently of WNK4.

In an attempt to define the role of WNK4 in the modulation of NCC in the face of changes in dietary K^+^ content, we studied the effects of low- and high-K^+^ diets in WNK4^−/−^ mice (4). The physiological parameters of WNK4^−/−^ mice maintained on low- and high-K^+^ diets were similar to those of wild-type mice, with the exception of plasma K^+^ concentration (Table 1). As previously reported (4), WNK4^−/−^ mice on a normal-K^+^ diet displayed mild hypokalemia (3.26 ± 0.5 vs. 3.99 ± 0.3 mM in wild-type mice, *P* < 0.01), which was aggravated when mice were fed a low-K^+^ diet (3.44 ± 0.6 vs. 2.03 ± 0.3 mM in wild-type mice, *P* < 0.00005). In contrast, the mild hypokalemia was corrected when mice were fed a high-K^+^ diet (Table 1).

**Fig. 5.**
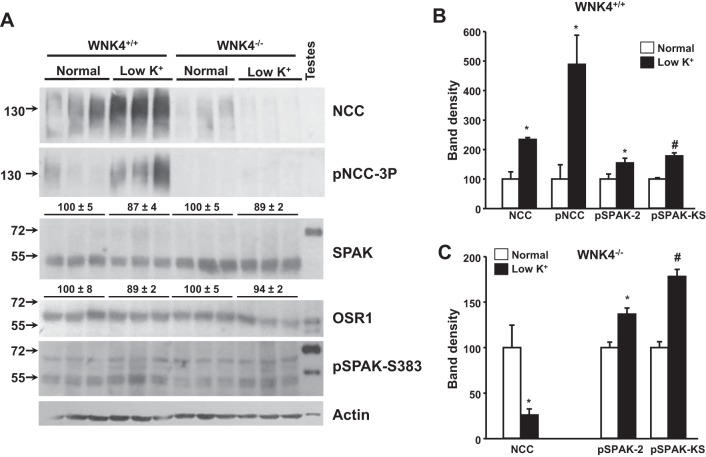
Effects of low-K^+^ diet on the expression and phosphorylation of Ste20-related proline-alanine-rich kinase (SPAK) and NCC in the renal cortex of with-no-lysine kinase 4 (WNK4)^+/+^ and WNK4^−/−^ mice. *A*: Western blot analysis of renal cortex protein samples of WNK4^+/+^ or WNK4^−/−^ mice kept on normal- or low-K^+^ diets. Representative blots are shown. Densitometric analyses were performed on at least two blots per assay, including samples of 6 mice/group. For NCC, pNCC, pSPAK2, and the kidney-specific form of pSPAK (pKS-SPAK), the results of these analyses are shown for WNK4^+/+^ mice (*B*) and WNK4^−/−^ mice (*C*). For the total SPAK blot, the top lines show densitometric results for KS-SPAK bands. OSR1, oxidative stress response 1 kinase. Results are expressed as mean percentages ± SE of the normal diet (100%). *P < 0.05; #*P* < 0.005 vs. the normal diet.

Western blot analysis of renal cortex protein extracts from WNK4^−/−^ mice fed either a normal- or low-K^+^ diet was performed in parallel to Western blot analysis of renal cortex protein extracts of WNK4^+/+^ mice. In this set of blots, increases in NCC expression and phosphorylation in response to a low-K^+^ diet were again observed in WNK4^+/+^ mice (Fig. 5, *A* and *B*). Analysis of pNCC behavior was difficult in WNK4^−/−^ mice because this colony exhibits low expression of the cotransporter and pNCC was undetectable in mice fed normal- and low-K^+^ diets (Fig. 5, *A* and *C*). However, SPAK and OSR1 analysis was possible. Total SPAK and OSR1 expression was unchanged by diet in both WNK4^+/+^ and WNK4^−/−^ mice (Fig. 5). T-loop phosphorylation was not studied because it could not be detected by Western blot analysis, as previously reported (25). SPAK S-motif (Ser^383^) phosphorylation levels increased in both WNK4^+/+^ and WNK4^−/−^ mice fed a low-K^+^ diet. This site was originally identified as a target for WNK phosphorylation (37), and we have shown that the signal observed with this antibody increases when SPAK is expected to be activated (4). Interestingly, this site is not only present in full-length SPAK but also in the shorter isoforms SPAK-2 and kidney-specific (KS-)SPAK (20).

**Fig. 6.**
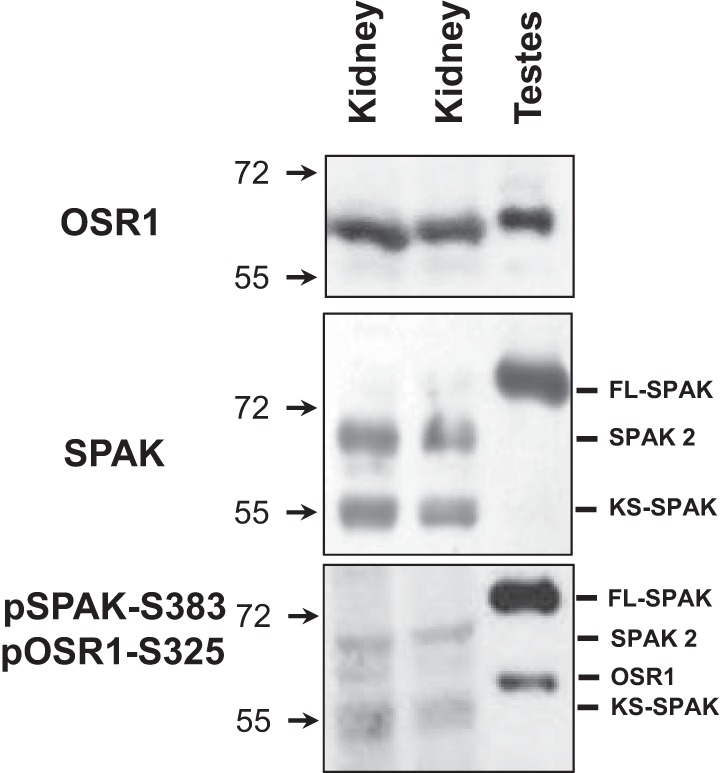
Characterization of the SPAK Ser^383^/OSR1 Ser^325^ phospho-antibody. Proteins extracted from mouse kidneys or testes were subjected to Western blot analysis with SPAK- and OSR1-specific antibodies and with a SPAK/OSR1 pSer^375^/Ser^325^ (S-motif) antibody. Labels on the right indicate to which protein each band corresponds according to the size and, in the case of the pSPAK/OSR1 blot, according to comparison with the other blots. FL-SPAK, full-length SPAK.

Although the SPAK peptide used to generate this phospho-antibody is very similar to the corresponding OSR1 peptide (25), based on the following evidence we inferred that, in the kidney, this antibody mainly recognizes SPAK. Using the SPAK-specific antibody, we detected two bands in kidney samples, whereas in the testes, a tissue in which full-length SPAK expression is abundant (25), a single, larger band was observed (Fig. 6). Thus, the two bands observed in the kidney samples with total SPAK antibody very likely correspond to SPAK-2 and KS-SPAK (20). In contrast, with the OSR1-specific antibody, a single band of similar size was observed in both kidney and testes samples. The size of this band was clearly different from the size of the SPAK bands. With the pSPAK Ser^383^ phospho-antibody, two bands were observed in the testes, one presumably corresponding to full-length SPAK and the other one to OSR1, given their size and given that short SPAK isoforms are not expressed in the testes (20, 25). In the kidney samples, two bands were also detected with the pSPAK Ser^383^ phospho-antibody. Because these two bands were different in size from those detected in the testes but similar to those observed with total SPAK antibody, we inferred that they most likely correspond to SPAK-2 and KS-SPAK. In support of this conclusion, in a recent study, Saritas et al. (30) observed that the signal detected in the kidney with this pSPAK Ser^382^ antibody was not observed in a SPAK knockout mouse in immunofluorescence experiments, not even in the presence of vasopressin, which stimulates SPAK S-motif phosphorylation in wild-type mice. Together, the blots shown in Fig. 6 and the data presented by Saritas et al. (30) suggest that the bands observed with the pSPAK Ser^382^ phospho-antibody in the kidney samples mainly correspond to SPAK2 and KS-SPAK. In the testes samples, however, pOSR1 was indeed observed.

In our study, the intensity of the SPAK-2 and KS-SPAK bands observed with this phospho-antibody was increased with the low-K^+^ diet in both WNK4^+/+^ and WNK4^−/−^ mice (Fig. 5). Thus, we concluded that phosphorylation of SPAK-2 and KS-SPAK increases in mice fed a low-K^+^ diet and that this effect is independent of the presence of WNK4. Consistent with previous reports (20, 25), we did not observe full-length SPAK in our blots of kidney samples. However, we believe that phosphorylation of this isoform may also be increased, but nevertheless remains undetectable by this technique due to the low levels of expression.

#### NCC phosphorylation induced by a low-K^+^ diet, but not ANG II infusion, is preserved in SPAK^T243A/T243A^ mice.

**Fig. 7.**
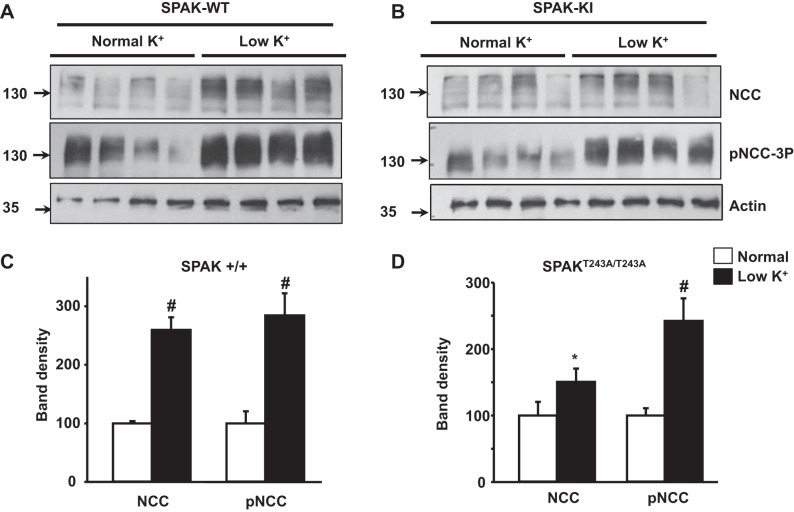
Effects of low-K^+^ diet on the expression and phosphorylation of NCC in SPAK^T243A/T243A^ mice. *A* and *B*: Western blot analyses of total kidney protein samples of SPAK^+/+^ [wild type (WT); *A*] and SPAK^T243A/T243A^ [knockin (KI); *B*] mice kept on normal- or low-K^+^ diets. Representative blots are shown. *C* and *D*: densitometric analyses of SPAK^+/+^ (*C*) and SPAK^T243A/T243A^ (*D*) mice for NCC and pNCC were performed on at least two blots per assay, including samples of seven mice for the normal-K^+^ groups and six mice for the low-K^+^ groups. Results are expressed as mean percentages ± SE of the normal diet (100%). *P < 0.05; #*P* < 0.005 vs. the normal diet.

SPAK^+/+^ and SPAK^T243A/T243A^ mice fed either a normal- or low-K^+^ diet had similar food intake, urinary Na^+^ excretion, and weight by the end of the study period (Table 2). Mice maintained on the low-K^+^ diet showed the expected reduction in urinary K^+^ excretion. As previously reported, no difference was observed in plasma K^+^ concentration between SPAK^+/+^ and SPAK^T243A/T243A^ mice on the normal diet, and both exhibited a decrease with the low-K^+^ diet. However, the difference between the values observed in the normal- versus low-K^+^ diets only reached significance in SPAK^T243A/T243A^ mice. Immunoblot analysis of total kidney proteins showed that total NCC expression and NCC NH_2_-terminal phosphorylation increased with the low-K^+^ diet in both SPAK^+/+^ and SPAK^T243A/T243A^ mice (Fig. 7). Thus, the response of NCC expression and phosphorylation to the low-K^+^ diet was present in mice expressing a catalytically inactive version of SPAK.

**Table 2.** Physiological parameters of SPAK^+/+^ and SPAK^T243A/T234A^ mice on NKD or LKD

	SPAK^+/+^ Mice		SPAK^T243A/T234A^ Mice	
	Means ± SE	*n*	Means ± SE	*n*
Food intake, g				
NKD	3.2 ± 0.3	6	2.6 ± 0.3	6
LKD	3.0 ± 0.2	6	2.4 ± 0.5	6
Water intake, ml				
NKD	7.3 ± 0.5	6	7.7 ± 0.9	6
LKD	8.8 ± 1.2	6	6.7 ± 1.0	6
Urinary volume, μl				
NKD	1.2 ± 0.2	6	1.4 ± 0.4	6
LKD	1.7 ± 0.6	6	1.3 ± 0.6	6
Weight, g				
NKD	27.0 ± 0.5	6	26.6 ± 0.6	6
LKD	26.8 ± 0.6	6	26.2 ± 1.2	6
*Urine data*				
Urinary Na^+^, mmol/24 h				
NKD	0.14 ± 0.03	5	0.15 ± 0.01	5
LKD	0.15 ± 0.03	4	0.10 ± 0.02	6
Urinary K^+^, mmol/24 h				
NKD	0.56 ± 0.10	5	0.51 ± 0.05	5
LKD	0.02 ± 0.003†	4	0.02 ± 0.004†	6
*Plasma data*				
Na^+^, mM				
NKD	156.50 ± 1.87	6	157.33 ± 1.86	6
LKD	156.17 ± 2.40	6	156.17 ± 1.47	6
K^+^, mM				
NKD	3.08 ± 0.56	6	3.79 ± 0.55	6
LKD	2.47 ± 0.43	6	2.60 ± 0.56†	6

Values are presented as means ± SE; the number of animals per group (*n*) is also shown. SPAK, Ste20-related proline-alanine-rich kinase. Urine collected on *day 4* of the treatment period was analyzed.

† *P* < 0.05 vs. NKD (same genotype).

**Fig. 8.**
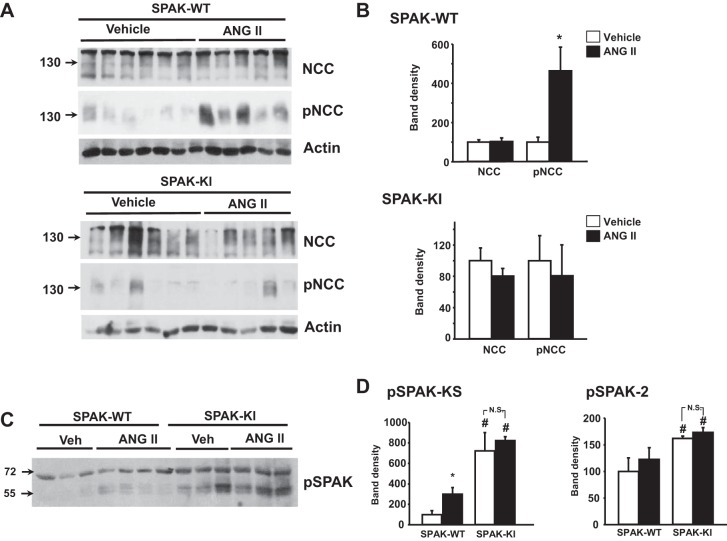
Effects of ANG II infusion on the expression and phosphorylation of SPAK and NCC in SPAK^T243A/T243A^ mice. *A*: Western blot analysis of NCC and pNCC in total kidney protein samples of wild-type SPAK and SPAK^T243A/T243A^ mice infused with vehicle or ANG II. *B*: densitometric analyses for NCC and pNCC were performed on at least two blots per assay, including samples of six mice for the wild-type groups and seven mice for the SPAK^T243A/T243A^ groups. *C*: Western blot analysis of pSPAK in total kidney protein samples of wild-type SPAK and SPAK^T243A/T243A^ mice infused with vehicle or ANG II. *D*: densitometric analysis of pKS-SPAK and pSPAK-2. *P < 0.05 vs. vehicle; #*P* < 0.05 vs. the wild-type group.

Given that the low-K^+^ diet-induced phosphorylation of NCC is preserved in SPAK^T243A/T243A^ mice, whereas the NCC phosphorylation induced by ANG II infusion is lost in WNK4^−/−^ mice (4), we decided to analyze the effect of ANG II infusion on NCC phosphorylation in the SPAK^T243A/T243A^ colony. Thus, SPAK^T243A/T243A^ mice and their corresponding wild-type controls were infused with ANG II for 4 days following the same protocol used for WNK4^−/−^ mice (4). Interestingly, as shown in Fig. 8, ANG II infusion resulted in a significant increase in NCC phosphorylation in wild-type mice, but this effect was not observed in SPAK^T243A/T243A^ mice. Additionally, ANG II infusion increased the phosphorylation of KS-SPAK in wild-type mice. This increase was not observed in SPAK^T243A/T243A^ mice, most likely because KS-SPAK phosphorylation was already increased in vehicle-treated animals. SPAK^T243A/T243A^ mice have been shown to develop a Gitleman's-like syndrome, with salt-remediable hypotension (25). These mice exhibit increased activity of the renin-angiotensin system, which causes increased KS-SPAK phosphorylation in vehicle-treated animals. However, the increase in KS-SPAK phosphorylation did not result in increased NCC phosphorylation, indicating that ANG II signaling through NCC in the DCT requires integrity of the WNK4-SPAK pathway (4, 29).

#### High-K^+^-citrate diet stimulates SPAK phosphorylation in both WNK4^+/+^ and WNK4^−/−^ mice.

WNK4^−/−^ mice maintained on a high-K^+^ diet for 4 days exhibited results similar to WNK^+/+^ mice. The high K^+^ content in the diet did not affect food consumption (Table 1). As expected, the plasma aldosterone concentration and urinary K^+^ excretion were significantly increased. The plasma K^+^ concentration remained within the physiological range, and, as previously reported, the difference in plasma K^+^ between WNK4^+/+^ and WNK4^−/−^ mice was no longer observed (4). In addition, as previously observed with high-K^+^ diets (7), the urinary volume and water consumption were significantly increased in both genotypes.

**Fig. 9.**
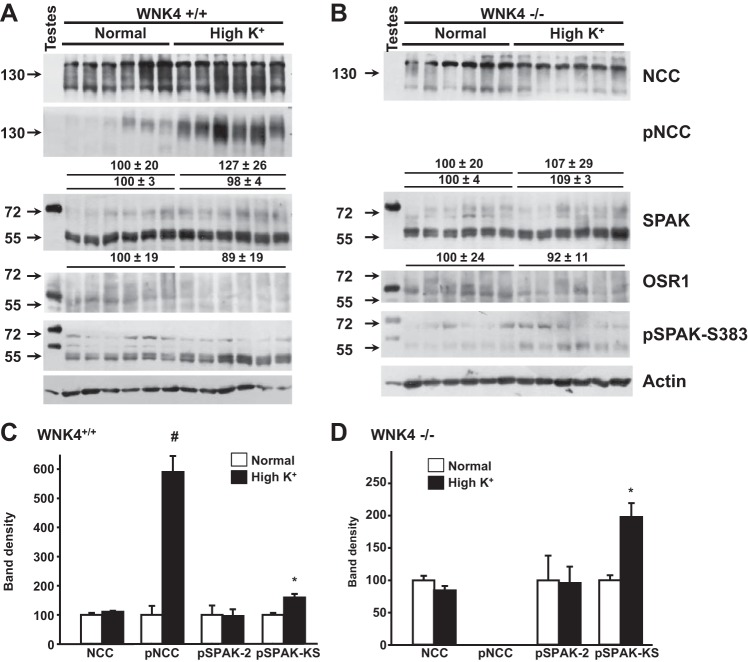
Effects of high-K^+^ diet on the expression and phosphorylation of SPAK and NCC in WNK4^−/−^ mice. *A* and *B*: Western blot analyses of total kidney protein samples of WNK4^+/+^ (*A*) or WNK4^−/−^ mice (*B*) kept on normal- or high-K^+^ diets. Representative blots are shown. *C* and *D*: densitometric analyses were performed on at least two blots per assay, including samples from six different mice per group, and are shown for WNK4^+/+^ mice (*C*) and WNK4^−/−^ mice (*D*). Results are expressed as mean percentages ± SE of the normal diet (100%). *P < 0.005; #*P* < 0.00005 vs. the normal diet.

With regard to the expression and phosphorylation of renal proteins, similar to what is shown in Fig. 1, in this new set of animals, we also observed that the high-K^+^-citrate diet promoted increased NCC phosphorylation in WNK4^+/+^ mice (Fig. 9). No pNCC was detected in WNK4^−/−^ mice, even when they were fed the high-K^+^ diet. Total NCC expression was unchanged by the high-K^+^ diet in WNK4^+/+^ and WNK4^−/−^ mice. SPAK and OSR1 total expression were also unchanged, but phosphorylation of the SPAK S-motif was significantly increased in both genotypes.

#### Activation of NCC by the high-K^+^ diet is aldosterone dependent.

**Fig. 10.**
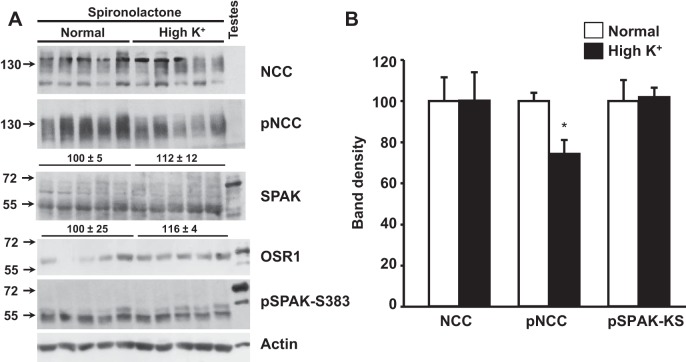
Effects of high-K^+^ diet on NCC and SPAK phosphorylation are blocked by spironolactone. Wild-type mice were kept on normal or high-K^+^ diets. Ethanol-dissolved spironolactone or vehicle (ethanol) was added to the drinking water. The calculated dose was 40 mg·kg^−1^·day^−1^. *A*: Western blot analysis of total kidney protein samples of wild-type mice on normal- or high-K^+^ diets treated with spironolactone. Representative blots are shown. *B*: Densitometric analyses were performed on at least two blots per assay, including samples from five different mice per group. Results are expressed as mean percentages ± SE of the normal diet (100%). *P < 0.05 vs. the normal diet.

It has been previously described that aldosterone stimulates NCC expression, phosphorylation, and, thus, activation (16, 35). Because the plasma aldosterone concentration is greatly increased in mice fed a high-K^+^ diet, we hypothesized that the increased NCC phosphorylation could be an aldosterone-induced effect. To investigate this hypothesis, we treated mice fed with either normal- or high-K^+^-citrate diet with the mineralocorticoid receptor blocker spironolactone. Interestingly, in spironolactone-treated mice, the high-K^+^ diet-induced increase in NCC and SPAK phosphorylation was not observed (Fig. 10), suggesting that these effects were indeed mediated by aldosterone. In contrast, NCC phosphorylation levels were reduced with the high-K^+^ diet in spironolactone-treated mice.

## DISCUSSION

Modulation of NCC expression/phosphorylation by changes in dietary K^+^ content has been previously reported (10, 34). In this study, we began to investigate the signaling pathways involved in this modulation, which are completely unknown, and we challenged certain previously made observations. Here, we showed that a low-K^+^ diet not only induced an increase in NCC NH_2_-terminal phosphorylation but also in NCC total expression. In addition, we showed that a low-K^+^ diet also increases the phosphorylation of SPAK-2 and KS-SPAK, the shorter isoforms of SPAK that are known to be expressed in the kidney (20). As previously described (25), in our blots, the full-length form of SPAK was not detected due to the low expression of this isoform in the kidney. However, it is accepted that, although undetectable by Western blot analysis, T-loop phosphorylation (Thr^243^) of this isoform occurs and is essential for SPAK activity within the kidney (25). In addition, although the relevance of S-motif phosphorylation is currently not as clear as that of T-loop phosphorylation, it has been shown to occur in conditions in which SPAK is expected to be activated to promote NCC function (4, 9, 35). The relevance of SPAK-2 and KS-SPAK phosphorylation in this and other situations remains unclear, given that these isoforms are predicted to be catalytically inactive (20). Anyhow, their S-motif is likely to become phosphorylated under the same stimuli that promote phosphorylation of this site in full-length SPAK. Thus, changes in the phosphorylation levels of these isoforms may be indicative of the activation state of the pathway, and here we are foreseeing that they are probably paralleled by changes in full-length SPAK phosphorylation. Thus, our data suggest that full-length SPAK is activated by low K^+^ intake and is responsible for NCC phosphorylation. This, however, should be carefully considered until further investigations clarify the roles of SPAK short isoforms' phosphorylation.

OSR1 expression was indeed detected in the kidney. However, as previously reported (20), OSR1 could not be detected with pSPAK/OSR1 S-motif antibody, which was capable of detecting phosphorylated OSR1 in the testes (Fig. 6). Thus, OSR1 S-motif phosphorylation levels appear to be very low in the kidney. Nevertheless, the low-K^+^-induced increase in NCC phosphorylation in SPAK^T243A/T243A^ mice, which lack SPAK catalytic activity due to impaired T-loop phosphorylation, suggests that another kinase is responsible for this phosphorylation. A strong candidate for this phosphorylation is OSR1. It has been recently shown that in SPAK-deficient (SPAK^−/−^) mice, OSR1 is upregulated in the DCT (30) and that OSR1 T-loop phosphorylation induced by vasopressin infusion occurs, suggesting that in SPAK^−/−^ mice, OSR1 is responsible for the vasopressin-induced increase in NCC phosphorylation. Another piece of evidence suggesting that, in the absence of SPAK in the DCT, OSR1 can mediate NCC phosphorylation is that crossing WNK4^D561A/+^ mice with SPAK^−/−^ mice does not completely prevent the pseudohypoaldosteronism type II phenotype, whereas crossing WNK4^D561A/+^ mice with SPAK^−/−^ mice and OSR1^+/−^ mice more efficiently reverts the phenotype (8). Thus, it is likely that NCC activation by a low-K^+^ diet in SPAK^T243A/T243A^ mice is due to a compensatory activity of OSR1.

The signaling pathway by which ANG II and a low-K^+^ diet increased NCC phosphorylation seems to be different. We have previously shown that SPAK-NCC phosphorylation induced by ANG II does not occur in WNK4^−/−^ mice (4). In this study, we show that the effect of ANG II on NCC phosphorylation is also lost in SPAK^T243A/T243A^ mice, confirming that WNK4-SPAK pathway integrity is required for ANG II signaling to NCC. Additionally, we observed that phosphorylation of SPAK in SPAK^T243A/T243A^ mice was already increased under basal conditions and that it was not further increased by ANG II. This could be due to the fact that in these mice, which are known to be salt depleted, a higher basal activity of the renin-angiotensin system is expected, which would probably promote higher basal levels of SPAK phosphorylation. Indeed, we have previously shown that SPAK S-motif phosphorylation is stimulated by a low-salt diet or ANG II infusion (4). This phosphorylation, however, cannot be translated into NCC activation because the T-loop site Thr^243^ cannot be phosphorylated and, thus, SPAK remains inactive (25). The observation that in SPAK^T243A/T243A^ mice ANG II infusion is not translated into NCC phosphorylation suggests that OSR1 activity cannot compensate for the loss of SPAK activity under this circumstance. In contrast, the positive effect of the low-K^+^ diet on SPAK phosphorylation persisted in WNK4^−/−^ and SPAK^T243A/T243A^ mice. Thus, WNK4 is not essential for SPAK phosphorylation under a low-K^+^ diet, as opposed to what was observed for activation by ANG II, and the absence of SPAK activity in SPAK^T243A/T243A^ mice is most likely compensated by OSR1 activity. It is possible that another WNK (namely, WNK1 or WNK3) may be responsible for SPAK/OSR1 activation when mice are fed a low-K^+^ diet. In this regard, it is worth noting that WNK3 has been shown to induce both NCC activation (12, 27, 39) and ROMK inhibition (18), making it a suitable kinase to promote increased salt reabsorption and reduced K^+^ secretion. Interestingly, although low-K^+^ diet-induced SPAK phosphorylation was observed in WNK4^−/−^ mice, NCC phosphorylation was not observed. These results suggest that, in the absence of WNK4, SPAK and NCC phosphorylation are uncoupled. The reason for this phenomenon is currently unknown. In addition, in the absence of WNK4, a decrease in total NCC expression in mice fed a low-K^+^ diet was observed, in contrast to the increase observed in wild-type mice. This finding may be due to the low aldosterone levels in these mice. Aldosterone is a well-defined positive modulator of NCC expression, and, in the absence of the positive stimulus (due to uncoupled SPAK-NCC phosphorylation), the aldosterone effect may be dominant.

Regarding NCC modulation under high dietary K^+^ intake, our results clearly contrast with those previously reported by others (10, 34). We observed that mice maintained on a high-K^+^-citrate diet had increased levels of pNCC, whereas in previous works, Vallon et al. (34) observed no changes or moderate decreases in pNCC, and Frindt and Palmer (10) reported decreased NCC apical expression. In this last work, K^+^ was added to the diet as KCl. Therefore, the high Cl^−^ intake might have induced the decrease in NCC surface expression, masking the effects of K^+^. There is no mention of the type of K^+^ supplementation used by Vallon et al. (34), but it was presumably also KCl. In the present study, K^+^ was added to the diet as K^+^-citrate while the Cl^−^ and Na^+^ dietary contents were kept constant to avoid confounding the effects of the different NCC modulators. Only in a selected experiment was K^+^ added to the diet as KCl to confirm the results shown by others. Indeed, we observed a decrease in the expression and phosphorylation of NCC with this diet. In addition, because the mice with the high-K^+^-citrate diet developed metabolic alkalosis, we performed experimental maneuvers to rule out that activation of NCC was due to the metabolic alkalosis produced by the high-K^+^ diet or by citrate itself. In wild-type mice, in which metabolic alkalosis was induced by HCO_3_^−^ loading, or in mice that were exposed to high citrate intake in the absence of increased K^+^ intake, no increase in NCC expression and phosphorylation was observed. Although rats exposed to citrate alone ingested, on average, 30% less amount of citrate compared with those exposed to K^+^-citrate, they still ingested an excess of citrate, and pNCC was not affected. Thus, neither metabolic alkalosis nor high citrate ion intake affect NCC phosphorylation. Additionally, we observed that increased NCC phosphorylation induced by the high-K^+^-citrate diet was prevented by spironolactone, indicating that it is dependent on mineralocorticoid receptor activation by aldosterone, which is known to be increased by K^+^ ingestion. Furthermore, observations made by Vallon et al. (34) also support the hypothesis of NCC upregulation caused by aldosterone in mice fed a high-K^+^ diet. Although these authors observed that the high-K^+^ diet induced mild decreases in NCC and pNCC, these effects were exacerbated in SGK1 knockout mice, and, for some NCC phosphorylation sites, the decrease was only observed in SGK1 knockout mice and not in wild-type mice. Because SGK1 is the aldosterone-responsive kinase that mediates certain aldosterone-induced effects, including the modulation of NCC (1), the positive effect of aldosterone may have been lost in SGK1 knockout mice, exacerbating the negative effect on NCC caused by that experimental protocol. More recently, Sornesen et al. (32) showed that acute K^+^ loading in mice, achieved by administering K^+^ through a gastric gavage or by allowing free access to food after a fasting period, induced a rapid decrease in NCC phosphorylation, which was related to the observed natriuresis. However, the K^+^ effect was clearly independent of aldosterone, whereas in our study, the NCC phosphorylation induced by chronic administration of a high-K^+^ diet was aldosterone dependent, indicating that the mechanisms mediating the acute and chronic effects are completely different. Thus, pNCC is increased when a high-K^+^ diet is administered with citrate, whereas it is decreased when administered with Cl^−^, suggesting that the inconsistencies between works are probably due to the anion coadministered with K^+^ (24). These results suggest that high Cl^−^ intake may exert an inhibitory effect on NCC phosphorylation/activity. In this regard, it has been shown that NCC as well as Na^+^-K^+^-Cl^−^ cotransporter (NKCC)1 and NKCC2 (15a, 22a, 23) are modulated by intracellular Cl^−^ depletion. Thus, we speculate that changes in Cl^−^ intake may promote changes in extracellular Cl^−^ concentration that may eventually be translated in changes in intracellular Cl^−^ concentration. Although changes in plasma Cl^−^ concentration are not always observed in the face of changes in Cl^−^ intake, it is known that many physiological responses occur without an evident change in the blood levels of the physiological parameter to be modulated. Further investigation will be required to explore potential mechanisms by which high Cl^−^ intake is translated into a decreased pNCC-to-NCC ratio.

SPAK S-motif phosphorylation levels also increased with the high-K^+^ diet in both WNK4^+/+^ and WNK4^−/−^ mice, and phosphorylation was prevented by spironolactone. Thus, the aldosterone-induced NH_2_-terminal phosphorylation of NCC was most likely due to increased SPAK activity. WNK4 does not seem to be involved in this activation. Notably, KS-SPAK phosphorylation increased, but SPAK-2 phosphorylation did not, in contrast to what we observed with the low-K^+^ diet. The meaning of this differential regulation of the phosphorylation of SPAK isoforms remains to be determined but could underlie the modulation of SPAK and NCC phosphorylation in mice fed the high-K^+^ diet.

Although it has been previously proposed that decreased NCC activity under a high-K^+^ diet is important to allow increased Na^+^ and flow delivery to more distal nephron segments involved in K^+^ secretion (2), our data suggest that this effect is achieved despite increased NCC activity because urinary volume was greatly increased in our mice on the high-K^+^ diet, as previously reported (6). This observation suggests that inhibition of proximal nephron Na^+^ and water reabsorption mechanisms may be more important during high K^+^ intake. For instance, it has been previously reported that Na^+^ reabsorption decreases under a high-K^+^ diet in the proximal tubule (3) and in the thick ascending limb of Henle's loop (6, 33).

In conclusion, we propose that both low- and high-K^+^-citrate diets promote NCC phosphorylation related to SPAK activation and that, under the high-K^+^ diet, the effect is aldosterone dependent. On the low-K^+^ diet, SPAK activity is apparently redundant for NCC activation, and OSR1 may be able to compensate for the loss of SPAK. The effects of low- and high-K^+^ diets on SPAK phosphorylation were present in WNK4^−/−^ mice, suggesting that these effects are not exclusively dependent on WNK4 and that other WNKs may be involved. This contrasts with the essential activity that WNK4 and SPAK play in the ANG II-induced activation of NCC.

## GRANTS

This work was supported by Mexican Council of Science and Technology Grant 165815 and Wellcome Trust Grant 091415 (to G. Gamba). M. Castañeda-Bueno was supported by a scholarship from the Mexican Council of Science and Technology and obtained a PhD degree from the Biochemical Science PhD program of the Universidad Nacional Autónoma de México.

## DISCLOSURES

No conflicts of interest, financial or otherwise, are declared by the author(s).

## AUTHOR CONTRIBUTIONS

Author contributions: M.C.-B. and G.G. conception and design of research; M.C.-B., L.G.C.-P., L.R.-V., I.A.-G., N.V., and E.M. performed experiments; M.C.-B., L.G.C.-P., L.R.-V., I.A.-G., N.V., E.M., and G.G. analyzed data; M.C.-B., L.G.C.-P., L.R.-V., I.A.-G., E.M., and G.G. interpreted results of experiments; M.C.-B., L.R.-V., I.A.-G., E.M., and G.G. prepared figures; M.C.-B. and G.G. drafted manuscript; M.C.-B., L.G.C.-P., N.V., and G.G. edited and revised manuscript; M.C.-B., L.G.C.-P., L.R.-V., I.A.-G., N.V., E.M., and G.G. approved final version of manuscript.
